# Racial Disparities in Fertility Care: A Narrative Review of Challenges in the Utilization of Fertility Preservation and ART in Minority Populations

**DOI:** 10.3390/jcm13041060

**Published:** 2024-02-13

**Authors:** Alexis K. Gadson, May-Tal Sauerbrun-Cutler, Jennifer L. Eaton

**Affiliations:** 1 Shady Grove Fertility Center, Rockville, MD 20850, USA; 2 Division of Reproductive Endocrinology and Infertility, Department of Obstetrics and Gynecology, Women and Infants Hospital and Warren Alpert Medical School of Brown University, Providence, RI 02903, USA; msauerbruncutle@wihri.org (M.-T.S.-C.); jleaton@wihri.org (J.L.E.)

**Keywords:** fertility preservation, ART, IVF, oocyte cryopreservation, embryo cryopreservation, racial disparities

## Abstract

Significant ethnic and racial disparities exist in the utilization and outcomes of assisted reproductive technology (ART) in the United States. The popularity of fertility preservation (FP) procedures, a specific application of ART for those desiring to delay childbearing, has increased; however, many minority populations have seen a less rapid uptake of these services. Minority patients pursuing ART are more likely to have poorer in vitro fertilization (IVF) and pregnancy outcomes. These outcomes are used to predict success after FP and may lessen the appeal of such procedures in these populations. Suboptimal outcomes are further compounded by challenges with receiving referrals to, accessing, and paying for FP services. Resolving these disparities in minority populations will require culturally appropriate education surrounding the benefits of ART and FP, the demonstration of favorable outcomes in ART and FP through continued research engaging minority participants, and continued advocacy for expanded access to care for patients.

## 1. Introduction

Assisted reproductive technology (ART) is any treatment or procedure that involves manipulating oocytes (eggs) and sperm to help a woman become pregnant, most commonly through in vitro fertilization (IVF) [1]. Since the first birth resulting from in vitro fertilization (IVF) in 1978 [2], ART has become more complex, is more widely available to patients in the United States, and its outcomes have significantly improved. Minority populations, however, have faced challenges in accessing care, and improvements in outcomes after treatment often lag those of their non-minority counterparts despite these advances. Merkison et al. recently performed a systematic review demonstrating that minority women have less access to insurance coverage for fertility care, longer durations of infertility prior to presenting to a specialist, lower rates of receiving counseling about fertility preservation before cancer treatment, and lower live birth rates after the use of ART [3].

Fertility preservation (FP), a newer application of ART, aims to save or protect oocytes, sperm, or reproductive tissue so that a person can use them to have biological children in the future [4]. FP can be pursued for a variety of reasons and provides flexibility for patients to build their family. FP has become increasingly popular, resulting in more coverage in the lay media and growing patient interest in this service. While utilization of these procedures has increased overall, minority populations have exhibited a slower trend in pursuing these services [5]. Despite increasing knowledge about fertility preservation procedures amongst provider and patient populations along with support for FP from the American Society for Reproductive Medicine (ASRM), racial and ethnic disparities persist in the utilization of FP.

The causes of the disparities in ART, FP utilization, and outcomes in minority populations are not well understood. Minority populations are underrepresented in ART and FP research, may lack the reproductive health knowledge necessary to pursue these services, and are often less likely to have easy access to these treatments [6]. According to Jackson-Bey et al., addressing these shortcomings and working to reduce provider bias in the health system are critical steps in the path to reducing racial and ethnic disparities in fertility care [6]. In this narrative review, we will explore racial and ethnic disparities in the utilization and outcomes of ART in minority populations in the United States with a specific focus on challenges in the uptake of fertility preservation in these groups.

## 2. Planned Oocyte Cryopreservation

Planned oocyte cryopreservation (OC) is egg freezing for future use by patients who choose to delay childbearing for a multitude of reasons other than imminent gonadotoxic treatments [7]. Alternatively, embryos can be created and cryopreserved for future use if a sperm source is available at the time of presentation. For many patients, the ability to cryopreserve oocytes provides the flexibility to create embryos with a partner or sperm donor in the future, thus keeping their reproductive options as broad as possible. Planned OC can be pursued for a variety of personal reasons including delaying child bearing for career advancement, current lack of a partner, insufficient funds for child rearing, or to preserve fertility in the case of a long-term illness that may shorten the reproductive lifespan [7,8].

Until 2012, oocyte cryopreservation was considered an experimental procedure by the American Society for Reproductive Medicine (ASRM) [9]. Since the removal of this label, the use of this technology has exponentially increased, and multiple studies have been published demonstrating its efficacy. A practice guideline from the ASRM dedicated to planned OC determined that there are insufficient data to determine the expected live birth rate after planned OC [10]. However, multiple studies show positive outcomes, particularly in patients who were younger at the time of their planned OC. Though retrospective in nature, these studies demonstrate that viable embryos can be created and live birth can be achieved after the thawing of previously cryopreserved oocytes [11,12,13,14,15,16,17]. Together, these studies demonstrate that planned OC and subsequent thawing are viable options for achieving a live birth.

### 2.1. The Reproductive Lifespan

Planned OC allows patients to “pause” ovarian aging by preserving oocytes at younger ages when the health of the oocytes is more optimal. The length of the reproductive lifespan is not infinite, and it is dictated by the health and size of the pool of oocytes within the ovary. Briefly, the development of the oocyte pool begins early in the fetus. The oocyte pool is largest (~5 million oocytes) between 16–20 weeks gestation, and from this point, irreversible atresia of the oocytes occurs. This atresia is paused from birth until puberty, when a cohort of oocytes is primed with each cycle and all but one oocyte undergoes atresia. At menopause, the oocyte pool is nearly depleted, with less than 1000 oocytes remaining from the millions that were initially stored in the ovary. In addition to the depletion of the number of oocytes in the ovarian pool, the quality of the remaining oocytes decreases over time, and the likelihood of successful spontaneous pregnancy also decreases [18]. After age 35, pregnancies are more likely to be chromosomally abnormal and lead to miscarriage or the live birth of neonates with chromosomal abnormalities such as Down syndrome. Because of these risks, planned OC is an excellent option to preserve young, healthy oocytes for utilization in the future when reproductive outcomes may be less favorable.

### 2.2. Racial and Ethnic Disparities in Planned OC Utilization

There are very few data focused on assessing racial and ethnic differences in accessing and utilizing planned OC in minority patients. A recent study using Society for Assisted Reproductive Technologies Clinic Outcome Reporting System (SART CORS) data showed that rates of planned OC between 2012 and 2016 increased amongst all demographic groups, regardless of ethnic background. Despite this uptick, planned OC remained underutilized by most ethnic minority populations, with only 33.5% of cycles during this period occurring in racial/ethnic minorities [5]. Notably, SART studies are generally limited by lack of complete and accurate race and ethnicity data. Approximately one-third of cycles within the database lack these data, and this often complicates analyses of the role of race/ethnicity in understanding outcomes.

The etiology of the disparities in the uptake of planned oocyte or embryo cryopreservation is unclear. However these disparities can be reasonably attributed to barriers that prevent the pursuit of any fertility treatment, including perceived racism, lack of insurance coverage for these procedures, and ease of access to an IVF clinic [3,19,20]. Minority patients have been shown to be less likely to seek infertility treatment and be more likely to discontinue treatment [19]. In a study querying barriers to access to care for minority patients at an academic center in Illinois, Black and Hispanic women were more likely to perceive race, weight, and income as direct barriers to accessing fertility care in comparison to White and Asian participants in the study. This study also demonstrated that Black and Hispanic patients may travel twice the distance to their IVF clinic in comparison to White and Asian patients [20]. Hispanic women have reported a higher perceived stigma surrounding the use of fertility services, and they may have more religious and ethical concerns regarding use of fertility treatments [21]. These studies indicate that a strong cultural influence surrounds the use of any fertility services, and that this influence likely extends to planned OC. These perceptions must be overcome to increase the utilization of these procedures.

### 2.3. Predicting Future Success after Planned OC in Minority Patients

Counseling patients regarding success rates after the use of previously cryopreserved oocytes can be challenging. Not only must cryopreserved oocytes be successfully thawed, the efficiency of fertilization and the likelihood of a successful live birth are crucial parameters used to appropriately counsel patients on expected outcomes when returning to use cryopreserved oocytes and embryos. Furthermore, the outcomes for patients undergoing planned OC for future autologous use cannot be compared to those for donor OC as donor oocytes are derived from younger women whose reproductive potential is presumed to be higher than that of planned OC patients, who often present at older ages.

Notably, studies specifically examining patients who have undergone planned OC have a low return rate, and outcomes are thus more challenging to assess [11,12,13]. Blakemore et al. performed a study assessing the likelihood of patients returning to utilize oocytes or embryos frozen during planned cryopreservation cycles. They specifically looked at return 10–15 years after initial cryopreservation, when disposition was most likely to be determined. At 10–15 years after cryopreservation, close to 60% of patients had not utilized their frozen oocytes and embryos. Of the patients who did return, most returned with a partner, regardless of their initial age at freezing [12]. Walker et al. demonstrated a similar no-return rate of 58.9% in patients who underwent planned and medical OC [11]. Leung et al. predicted a much more modest return rate of 15.6%, with most patients returning to utilize cryopreserved oocytes within 4 years of cryopreservation [13]. These low return rates can be attributed to the fact that planned OC is considered an insurance policy against reproductive aging and may not be required for family building. These studies propose that the low return rate results from some patients spontaneously conceiving and not requiring their cryopreserved oocytes, some patients simply choosing not to start a family, and, importantly, too little time elapsing after freezing for patients to return and use cryopreserved oocytes and embryos [11,12,13].

Success rates after oocyte thaw and insemination, therefore, must be extrapolated from predicted success after IVF cycles for infertility. A recent study utilizing SART data collected between 2014 and 2016 demonstrated a significantly lower live birth rate in Black patients in comparison to White and Asian patients, though not when compared to Hispanic patients. The implantation rate was significantly lower and the miscarriage rate was significantly higher when comparing Black patients to White patients. The authors further analyzed cycles which utilized pre-implantation genetic testing for aneuploidy (PGT-A), a technology in which embryos are biopsied to determine the chromosomal makeup of the embryo and preferentially select only euploid (chromosomally normal) embryos for transfer to decrease the risk of miscarriage and shorten the time to live birth. Despite the utilization of this technology, the authors noted a significantly lower implantation rate in Black patients when compared to other ethnic groups [22]. A review by Huddleston et al. further examined inequities in medically assisted reproduction and found lower live birth rates among multiple minority groups (Black, Asian, Middle Eastern/North African). These groups were additionally challenged by high miscarriage rates, low implantation rates, lower fertilization rates, and higher likelihoods of preterm or early preterm delivery [19].

The reasons for the lower rate of IVF success in minority groups are not well understood, but they are consistently demonstrated in the literature. The data demonstrate suboptimal outcomes for minority patients in ART cycles; patients may be understandably deterred from pursuing planned OC given the uncertainty of favorable outcomes when returning to utilize cryopreserved gametes.

### 2.4. Systemic Bias and Racism in Medicine

The previously mentioned suboptimal outcomes in minority populations are compounded by a persistent underlying mistrust of the medical system in minority communities. The dark history of the medical mistreatment of minority groups in the United States has demonstrated that advancements in medicine and the utilization of novel technology can easily come at the detriment of the health of minority populations through a lack of informed consent and the non-disclosure of potentially beneficial treatment. From the development of instruments and procedures trialed on the slaves Anarcha, Lucy, and Betsey to the Tuskegee experiment, the contraceptive trials in Puerto Rico, and the use of cells derived from Henrietta Lacks that revolutionized our ability to grow cells in culture, many major medical advancements in the United States have been derived from knowingly unethical experimental trials in minority communities. This long history has far-reaching implications for modern healthcare, including hesitancy to enroll in research studies and skepticism around experimental procedures. Minority patients may be more skeptical of planned OC, particularly as oocyte cryopreservation was considered an experimental procedure shortly before its rapid acceptance and uptake in society. Serious questions about autonomy over frozen tissue, the use of any frozen tissues for research, and the overall safety of the procedures may arise in minority patients considering fertility preservation. Ultimately, widespread, culturally sensitive education about planned OC and increased efforts to reduce out-of-pocket cost for patients will be required to decrease disparities in the utilization of this treatment.

## 3. Fertility Preservation Prior to Gonadotoxic Therapies

Fertility preservation in the form of oocyte or embryo cryopreservation can also occur prior to gonadotoxic therapies such as chemotherapy, radiation, or other procedures that may cause infertility [23]. It is estimated that more than 86,000 Americans between the ages of 15 and 39 received a new cancer diagnosis in 2023. Approximately 86% of them will survive 5 years after diagnosis and will question their desire to build a family after receiving treatment [24]. Importantly, despite improved diagnostic techniques and treatments, minority patients are more likely to have delayed cancer diagnoses and are more likely to be diagnosed at an advanced stage, which can necessitate expedited treatment and reduce the time available for fertility preservation procedures [25].

The decision to pursue fertility preservation adds an additional layer of complexity and nuanced decision-making for families facing a devastating diagnosis. According to ASRM, fertility preservation should be offered to all patients facing potentially gonadotoxic treatment [26]. However, this recommendation is far from universally applied to patients who receive a cancer diagnosis. Furthermore, minority patients are even less likely to receive adequate counseling and a referral for FP procedures. A recent study by Voigt et al. comparing the racial/ethnic background of the population of cancer patients in New York City to that of patients presenting for fertility preservation counseling showed that a lower-than-expected proportion of patients identified as minorities. Minority patients were older, more likely to be partnered, and more likely to undergo embryo-banking fertility preservation cycles [27]. Others have posited that race does not influence referral for or pursuit of fertility preservation. However, these studies are often performed in academic centers where interdisciplinary referrals and close follow ups may be more easily facilitated [28,29]. Voigt et al. additionally assessed sociodemographic differences in the utilization of fertility services and found that even when referred, Hispanic and non-Hispanic Black women were less likely to utilize fertility preservation services [30].

### 3.1. Lack of Referrals and Counseling

Oncologists play a crucial role in assessing patients’ desire for fertility preservation at the time of diagnosis and are often the first providers to engage in the fertility preservation conversation. Patients are much more likely to pursue fertility preservation if they are referred prior to undergoing treatment, yet fewer than half of surveyed providers refer patients of reproductive age to fertility specialists [28]. Data regarding the role of race/ethnicity in referrals for FP procedures have been conflicting. Goodman et al. demonstrated that non-White women were fifty percent less likely to be referred for fertility preservation counseling than White patients [31]. A more recent study by Swain et al. performed across multiple Detroit area clinics demonstrated no significant difference in counseling or the utilization of FP services when comparing Black and White patients [32].

The disparities in referral are further magnified by differences in the counseling that patients receive. Multiple studies have shown that the documentation of discussions of infertility risk, fertility preservation counseling, and referral to an infertility specialist was significantly lower in patients older than 40 years and those with at least one child [32,33]. Those who did not obtain higher education, who were older, or who had previously had children were less likely to receive counseling [34]. Lawson et al. found that while the greatest predictor of receiving fertility preservation counseling was a cancer diagnosis, non-White patients were 20% less likely to be counseled regardless of their diagnosis [35]. Similarly, Goodman et al. found that patients who had not attained a bachelor’s degree or who already had children were less likely to be counseled regarding fertility preservation [31]. A phone survey of board certified providers who provide services at sites that serve at least 30% racial/ethnic minorities demonstrated that the time required to counsel patients about fertility preservation, cost (regardless of insurance coverage), age, and lack of access to required transportation were perceived barriers to the more universal referral of patients for fertility preservation counseling [36].

### 3.2. Role of Insurance in Reducing Disparities

Insurance coverage of fertility preservation services is largely seen as the next step to reducing healthcare disparities. In the past, patients without insurance were four times less likely to be referred for fertility preservation counseling [31]. As of January 2024, sixteen states have implemented insurance mandates for the coverage of fertility preservation care prior to gonadotoxic treatments [37]. There is a paucity of data on the effect that insurance mandates have had on referrals for fertility preservation. However, recent data from the Rhode Island Fertility Preservation Registry have shown that despite the increased uptake of fertility preservation services, almost all patients who ultimately underwent fertility preservation cycles carried commercial insurance [38]. A recent study showed that the vast majority of patients presenting for fertility preservation had some type of insurance, and this likely reflects referral bias toward patients who are likely to proceed with treatment as a result of lower out-of-pocket costs [27]. Correia et al. utilized SART CORS data comprised of over one million autologous IVF cycles and found that the implementation of an insurance mandate for infertility coverage resulted in increased utilization across all racial and ethnic groups. Despite increased insurance coverage in mandated states, minority groups still showed utilization comparable to that of patients in non-mandated states. The mandate did not impact clinical outcomes, and the live birth rate was lower in all groups of racial and ethnic minorities [39]. Korkidakis et al. performed a study using estimates from the US Census Bureau and the Centers for Disease Control and Prevention (CDC) to compare utilization amongst ethnicities in mandated versus non-mandated states. They found that minorities were least likely to utilize ART; however, that utilization was increased by the implementation of an insurance mandate. Additionally, the disparities further narrowed when assessing utilization amongst eligible patients [40]. These studies highlight the important role of insurance mandates in reducing disparities in the utilization of ART, but they demonstrate that eliminating disparities requires much more than an insurance mandate alone.

Overall, insurance mandates for fertility preservation and fertility services seem promising, but they are not without their flaws. It is important to acknowledge that while insurance mandates improve access for patients who have commercial insurance, a large percentage of reproductive-age women in the United States utilize publicly funded insurance and lack coverage for fertility preservation and other fertility-related services [41]. Furthermore, many commercial insurance plans do not cover fertility preservation services, even if infertility services may be covered. This lack of insurance coverage makes fertility preservation inaccessible for most reproductive-age women in the United States.

It is also important to acknowledge that access to insurance coverage for any fertility treatment is almost inextricably linked to employment in the United States. Recently, more companies have begun offering infertility and OC benefits to employees. These benefits make planned OC more financially feasible for patients. Mercer’s National Survey of Employer-Sponsored Health Plans in 2022 showed a steady increase in the number of employers offering fertility and OC benefits: up to 21% of survey respondents in 2021 [42]. Data also demonstrate that employees are more likely to pursue fertility preservation if a company benefit is available [43,44]. Multiple studies purport that insurance coverage alone cannot eliminate racial and ethnic disparities in ART usage. However, insurance coverage does increase the utilization of fertility preservation procedures. The intricacies of insurance and fertility preservation access are more fully explored in *The Status of Fertility Preservation (FP) Insurance Mandates and Their Impact on Utilization and Access to Care* by Sauerbrun-Cutler et al.

### 3.3. Streamlining Fertility Preservation Care

There are data that demonstrate that streamlining fertility preservation care may help to reduce disparities in the utilization of fertility preservation treatment prior to gonadotoxic therapies. Flink et al. observed that providing all patients with an informed fertility preservation consult regardless of insurance status increased the utilization of fertility preservation services to nearly 50% of patients. In their study, about 25% of patients had Medicaid insurance, and while this alone was not a significant factor in determining whether patients proceeded to treatment, race was [45]. Additionally, the establishment of an automatic prompt for fertility preservation counseling within the electronic medical record system has been shown to increase the number of patients counseled or referred to a fertility specialist [35].

## 4. Limitations and Future Directions

**Figure 1:**
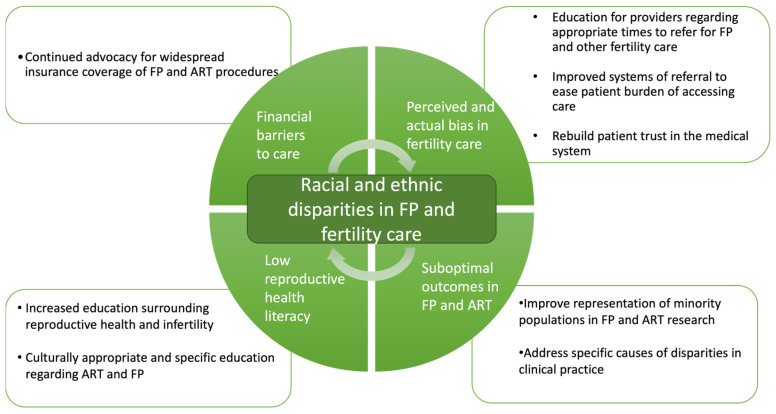
Proposed causes of and potential solutions to the observed racial and ethnic disparities in FP and fertility care in the United States.

There are many limitations in the current literature concerning the care of minority patients seeking fertility care. The barriers and potential solutions are summarized in Figure 1. Research into disparities, particularly in terms of race/ethnicity, are challenging as personal identity can be complex and not easily characterized by the options available to most patients in clinical settings. The categories available for race/ethnicity often do not fully reflect the diverse backgrounds of patients. Furthermore, race or ethnicity may be assigned by staff rather than identified by the patients themselves. Much of the available clinical data may be unreliable due to these flawed collection methods. Future data collection should focus on allowing patients to self-identify both race and ethnicity while broadening the breadth of options from which patients may choose. By doing so, future research may further pinpoint the cultural differences that contribute to attitudes toward ART and fertility preservation and influence outcomes amongst these groups.

Fertility preservation prior to gonadotoxic therapies presents a unique convergence of advocacy for access to fertility preservation services for all patients who desire them and the need for urgent medical treatment that may endanger their reproductive future. Despite widening access to fertility preservation when there is an urgent medical indication, disparities persist in the rates of referral and uptake of treatment. Institutions that have established interdisciplinary teams to facilitate referrals for all patients regardless of insurance and socioeconomic status have appeared to be more successful in reducing disparities. These programs should serve as templates for other centers aiming to serve these patients. Advocacy for insurance coverage for fertility preservation prior to fertility-threatening treatment is ongoing, and coverage is being codified in more states. This coverage should be further extended to patients who have publicly funded insurance to continue to remove the disparities in fertility preservation utilization in this population.

Additionally, work must continue to ease the distrust in the medical system that has been entrenched in minority and other marginalized populations for generations. The approach to alleviating this distrust will be multi-faceted and should include culturally sensitive education specific to minority populations, intentional community outreach, and the continued recruitment of minority patients into research studies.

## 5. Conclusions

General awareness of fertility preservation and ART for any indication has dramatically increased in recent years. Despite increasing knowledge, several racial and ethnic disparities exist and affect access to, utilization of, and outcomes of these services. Addressing the challenges that minority groups have faced requires addressing gaps in the knowledge of patients seeking these services in a culturally respectful manner, actively recruiting minority patients to participate in research that will further elucidate the causes of the known disparities, advocating for expanded insurance coverage for FP and ART, and repairing the relationships between minority communities and the medical system in the United States.

## Data Availability

No new data were created or analyzed in this study. Data sharing is not applicable to this article.
